# Synaptic Dysfunction in Dystonia: Update From Experimental Models

**DOI:** 10.2174/1570159X21666230718100156

**Published:** 2023-09-01

**Authors:** Ilham El Atiallah, Paola Bonsi, Annalisa Tassone, Giuseppina Martella, Gerardo Biella, Antonio N. Castagno, Antonio Pisani, Giulia Ponterio

**Affiliations:** 1 Laboratory of Neurophysiology and Plasticity, IRCCS Fondazione Santa Lucia, Rome, Italy;; 2 Department of System Medicine, University of Rome Tor Vergata, Rome, Italy;; 3 Department of Biology and Biotechnology “L. Spallanzani”, University of Pavia, Pavia, Italy;; 4 Department of Brain and Behavioral Sciences, University of Pavia, Pavia, Italy;; 5 IRCCS Fondazione Mondino, Pavia, Italy

**Keywords:** Synaptic dysfunction, movement disorders, dystonia, striatum, cerebellum, rodent models

## Abstract

Dystonia, the third most common movement disorder, refers to a heterogeneous group of neurological diseases characterized by involuntary, sustained or intermittent muscle contractions resulting in repetitive twisting movements and abnormal postures. In the last few years, several studies on animal models helped expand our knowledge of the molecular mechanisms underlying dystonia. These findings have reinforced the notion that the synaptic alterations found mainly in the basal ganglia and cerebellum, including the abnormal neurotransmitters signalling, receptor trafficking and synaptic plasticity, are a common hallmark of different forms of dystonia. In this review, we focus on the major contribution provided by rodent models of DYT-*TOR1A*, DYT-*THAP1*, DYT-*GNAL*, DYT/ PARK-*GCH1*, DYT/PARK-*TH* and DYT-*SGCE* dystonia, which reveal that an abnormal motor network and synaptic dysfunction represent key elements in the pathophysiology of dystonia.

## 1 INTRODUCTION

Dystonia is a movement disorder characterized by involuntary sustained or intermittent muscle contractions, resulting in repetitive and patterned movements and/or abnormal postures [1]. The dystonia definition comprises a heterogeneous group of etiologically distinct hereditary, acquired and idiopathic forms of a hyperkinetic disorder. Given the absence of significant neurodegeneration, dystonia is considered a functional disorder, characterized by neuronal network abnormalities in specific brain regions involved in motor control, including basal ganglia, thalamus, cerebellum and cerebral cortex [2, 3]. In isolated forms, dystonia is the only clinical feature, except for tremor, while in combined dystonia, other neurological signs, such as parkinsonism, myoclonus or chorea, are present [4, 5]. Although dystonia represents the third most common movement disorder that significantly affects the quality of life of patients and their families, to date, only symptomatic treatments are available [6]. Evidence from experimental and clinical studies suggests that synaptic dysfunction represents a key factor in the pathophysiology of dystonia. However, the exact cellular and molecular mechanisms driving the manifestation of dystonic symptoms remain to be fully clarified [2, 6]. Experimentalmodels of dystonia represent an invaluable tool to examine the neural network circuitry and the synaptic function, which is a fundamental step for the identification of novel therapeutic targets and/or specific biomarkers of disease progression [3, 7, 8]. In fact, etiologic and symptomatic models have been generated in order to reproduce the etiology (construct validity) and mimic the phenotype (face validity) of human dystonia [9, 10]. To date, 28 dystonia (DYT) loci have been linked to different genetic forms of dystonia [5, 11]. Of interest, many of these genes converge on common neurobiological pathways involved in synaptic transmission [12-15]. In the present review, we will focus on advances in the research on the synaptic function in dystonia obtained from experimental models of the most extensively studied forms of isolated dystonia: DYT-*TOR1A* (generalized early-onset dystonia); DYT-*THAP1* (craniocervical and limb dystonia); DYT-*GNAL* (adult-onset craniocervical dystonia), and of combined dystonia: DYT/PARK-*GCH1* and DYT/PARK-*TH* (dopa-responsive dystonia, DRD), and DYT-*SGCE* (myoclonus-dystonia).

## 2 SYNAPTIC DYSFUNCTION IN MOVEMENT DISORDERS

Synaptic dysfunction has been linked to a variety of neurological diseases, including epilepsy, autism spectrum disorders, psychiatric and movement disorders [16-18]. In particular, movement disorders share several molecular alterations that can lead to changes in synaptic plasticity [19]. In Parkinson’s Disease (PD), altered intracellular trafficking, lysosomal and mitochondrial impairment have a key role in generating dysfunction at the synapse, including loss of corticostriatal synaptic plasticity [20, 21]. In Huntington's disease (HD) the primary site of dysfunction is the corticostriatal synapse, where mutant huntingtin (htt) modifies the striatal excitatory synaptic activity by modulating N-methyl-d-aspartate receptor (NMDAR) signaling [22]. In dystonia, both preclinical and clinical evidence supports a significant disruption of homeostatic plasticity, characterized by facilitation of synaptic potentiation coupled with the loss of inhibitory processes [23-25]. Although most experimental models do not exhibit overt dystonia, they share a common endophenotype: an abnormal corticostriatal synaptic plasticity [19, 24, 26, 27]. Consistently, several genes linked to dystonia are involved in synaptic trafficking and neurotransmission [13, 14]. To date, additional evidence, driven by studies on genetic animal models, supports the idea that dystonia can be considered a synaptopathy, and that the alterations in synaptic function can represent a key event in its pathogenesis [15, 28].

## 3 CLINICAL AND EXPERIMENTAL EVIDENCE OF SYNAPTIC DYSFUNCTION IN DYSTONIA

Considering their role in motor control, the basal ganglia and the cerebellum are believed to represent key brain areas in dystonia pathophysiology. Although this topic is not the subject of the present review, for which we refer the readers to other more pertinent works [6, 29, 30], in this paragraph we will provide a brief overview of the synaptic dysfunction of these brain regions in dystonia (Tables **1**, **2**).

Clinical and pre-clinical research support the role of the cerebellum in the pathophysiology of dystonia [31, 32]. The cerebellum is involved in the control, coordination, and planning of voluntary movements [33] and is connected to the basal ganglia *via* the cortico-ponto-cerebello-thalamocortical loop and specifically to the nucleus striatum *via* the intralaminar thalamic nuclei [29, 34, 35]. More relevant, positron emission topography (PET) studies disclosed an increased cerebellar metabolic activity in dystonic patients, and cerebellar stimulation was able to alleviate dystonia symptoms in some of them [34, 36-38]. Moreover, repetitive transcranial magnetic stimulation (rTMS) of cerebellar hemispheres is under investigation as a potential therapy for primary cervical dystonia [39]. Cerebellar damage, caused by stroke or tumors, can determine the onset of dystonic movements [40-42], and preclinical studies have shown that manipulations such as the injection of kainic acid in the cerebellar cortex may induce dystonic-like postures in control mice [43]. Furthermore, genetic animal models of dystonia have reported subtle changes in Purkinje cells (PC) and cerebellar deep nuclei (DN) microarchitecture and activity in DYT-*TOR1A*, DYT-*THAP1*, and DYT-*SCGE* mouse models [44-49]. Recent work highlighted an aberrant cerebello-thalamic pathway in a DYT-*GNAL* mouse model, showing that a theta-burst stimulation of the cerebellar nuclei fails to induce potentiation of the cerebello-thalamic synapses [50].

Although the specific mechanism by which the cerebellum is involved in dystonia pathogenesis is still unknown, growing evidence supports its role in the generation of dystonic-like movements, in line with the concept of dystonia as a brain network disorder.

The basal ganglia have been held accountable for many years as the origin site of dystonia [51-53]. These subcortical nuclei include the striatum (caudate-putamen, CP, and nucleus accumbens, NAc), globus pallidus (GP), subthalamic nucleus (STN), and substantia nigra (SN), and are involved in motor control, procedural learning, executive function and emotion [54]. The striatum, the major input nucleus of the basal ganglia, receives glutamatergic afferents from both the cerebral cortex and thalamus [54]. The activity of the main population of striatal cells, the GABAergic projection neurons (SPNs), is additionally finely tuned by a highly regulated balance between acetylcholine, released from a small number of local interneurons (ChIs), and dopamine, originating from midbrain ventral tegmental area and SN terminals. Such balance is central for the control of motor activity [55]. Indeed, an abnormal dopaminergic transmission has been reported in dystonia patients and experimental models [52, 56, 57]. Further, clinical observations show that dystonia may occur in patients with PD, or as a consequence of treatment with dopaminergic drugs. However, only DYT/PARK-*GCH1* patients benefit from L-DOPA treatment. The other forms of dystonia usually do not respond to dopaminergic therapy, and benefit more from anticholinergic drugs, in particular trihexyphenidyl [39, 58, 59]. Accordingly, experimental data from preclinical models demonstrate cholinergic dysfunction in different forms of dystonia, suggesting that diverse molecular mechanisms may lead to common alterations [60-63].

PET imaging studies have clearly demonstrated that basal ganglia activity is affected in different forms of dystonia, showing, in particular, a reduction of the putamen-GPe connection, coupled with increased inhibition of STN and GPi [64, 65]. Interestingly, carriers of the DYT-*TOR1A* and DYT-*THAP1* mutations, either manifesting or non-manifesting dystonia, showed abnormalities in the cortico-striato-pallido-thalamo-cortical pathways, supporting the concept of dystonia as a circuit disorder [34, 36]. Dystonia patients show increased neuronal activity of the GPi, which is correlated with symptom severity and can be suppressed by Deep Brain Stimulation (DBS), further supporting the role of basal ganglia in dystonia pathophysiology [66, 67]. In particular, GPi-DBS has long-term efficacy in severe forms of primary generalized dystonia, such as DYT-*TOR1A* and DYT-*GNAL*, and may ameliorate the myoclonus and/or dystonia severity in the DYT-*SGCE* and DYT-*THAP1* patients [68-70].

## 4 SYNAPTOPATHY IN EXPERIMENTAL MODELS OF DYSTONIA

“Isolated” dystonia and dystonia plus syndromes are disorders characterized by neuronal defects involving several neurotransmitters [71, 72]. Proteins directly connected to synaptic function implicated in the genetic forms of dystonia like DYT-*TOR1A*, DYT-*THAP1*, DYT-*GNAL*, DYT/PARK-*GCH1*, DYT/PARK-*TH* and DYT-*SGCE* offer the opportunity to understand the pathophysiological mechanisms underlying the synaptic transmission imbalance (Tables **1**, **2**).

### 4.1 DYT-TOR1A Dystonia

**Fig. (1):**
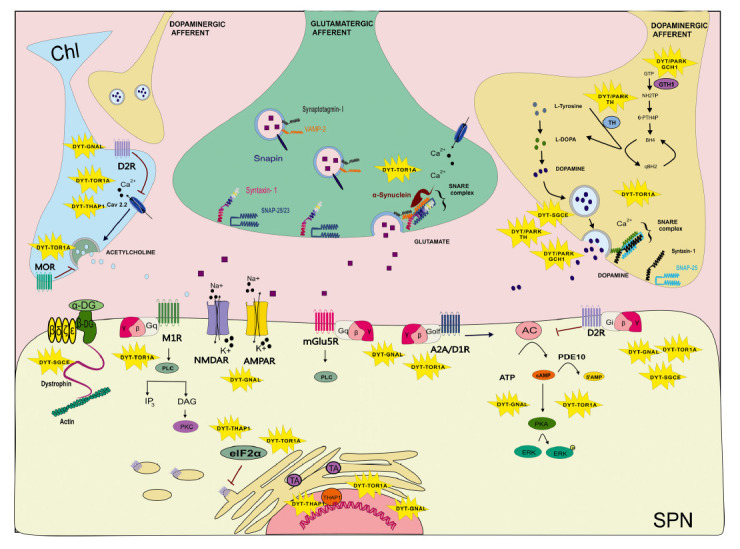
Schematic model of synaptic dysfunction in dystonia. Schematic overview of the role of dystonia genes implicated in striatal signaling. The proteins encoded by DYT genes (marked with yellow stars) are involved in synaptic transmission and may converge on common pathways. At pre-synaptic level: In DYT/PARK-*TH* and DYT/PARK-*GCH1* dystonia, mutations in tyrosine hydroxylase (*TH*) and GTP cyclohydrolase 1 (*GCH1*) result in reduced dopamine (DA) synthesis. Dystonia genes *SGCE*, *TOR1A*, *GCH1* are involved in dopamine release, while *TOR1A*, *GNAL* and *THAP1*, by inducing an abnormal D2R activation on cholinergic interneurons, affect acetylcholine release. DYT-*TOR1A* is also characterized by an enhanced mu opioid (MOR) neurotransmission. In addition, the AAA+ protein TorsinA (TA) (DYT-*TOR1A*) and its relationship with α-synuclein impairs glutamate release. At post-synaptic level: SGCE is a component of the dystrophin-glycoprotein complex that makes connections between the extracellular matrix and the intracellular actin cytoskeleton. In SPNs, mutations in the G_αolf_ protein (DYT-*GNAL*) and in the TA protein (DYT-*TOR1A*) alter the signal transduction of D1R, A2AR, D2R, affecting the cAMP signaling cascade. In addition, DYT-*TOR1A* and DYT-*GNAL* affect glutamatergic and muscarinic transmission. Dystonia genes *TOR1A* and *THAP1* are involved in the stress response, acting on eIF2α and secretory pathways in the endoplasmic reticulum, and together with *GNAL,* influence the gene transcription.

DYT-*TOR1A* is caused by a 3-bp deletion (GAG) in exon 5 of the *TOR1A* gene, resulting in the loss of a glutamic acid residue in the C-terminal of the TorsinA (TA) protein (ΔE-TA) [73]. Due to the homology between the human *TOR1A* and the rodent *Tor1a* genes, and the availability of multiple animal models, the early-onset DYT1 dystonia (Oppenheim’s dystonia) is the most extensively studied. These animal models of DYT1 dystonia, including knockin (KI), knockout (KO) and conditionals of *TOR1A*, represent a fundamental tool to understand the mechanisms by which mutated TA disrupts neuronal homeostasis. TA, a member of the AAA+ superfamily of ATPases, typically acts as a chaperone, to mediate the conformational changes of target proteins in the endoplasmic reticulum (ER), where it is more abundantly expressed [74]. Additionally, ultrastructural studies have shown that striatal TA can associate with small vesicles at presynaptic terminals, supporting the role of TA in the recycling and release of synaptic vesicles (Fig. **1**) [75, 76]. *In vitro* studies have demonstrated that TA interacts with snapin, an important component of the vesicle exocytosis machinery, and with vesicle-associated membrane protein 2/synaptobrevin (VAMP-2), a marker of synaptic vesicles [77]. Therefore, TA may act as a chaperone also at the synaptic level, affecting the synaptic vesicle turnover and, consequently, the neurotransmitter release [28]. Indeed, our recent work has shown a novel and critical relationship between alpha-synuclein and TA, affecting some synaptic proteins of the SNARE complex and glutamate release (Fig. **1**) [15], and suggesting synaptic modulators as a potential therapeutic approach [6]. TA is enriched in striatal ChIs and cerebellar PCs [78]. Electrophysiological studies in the DYT1 mouse striatum have provided evidence for impairments of multiple neurotransmitter systems [79-83] and loss of the balance between dopamine and acetylcholine. The *Tor1a*^+/Δgag^ KI mice, carrying the trinucleotide GAG deletion within the endogenous murine *Tor1a* gene, and the *Tor1a*^+/−^ null (KO) mice [84] recapitulate some of the alterations described in patients, such as a reduced striatal dopamine release and D2R binding and level, in addition to an increased cholinergic tone and an impaired corticostriatal synaptic plasticity [27, 63, 85, 86]. In multiple models, activation of dopamine 2 receptor (D2R) induces a paradoxical excitatory, rather than inhibitory, response of striatal ChIs [80, 85, 87-90]. This abnormality produces an excessive release of acetylcholine [63, 90], and the overactivation of muscarinic type 1 receptors (M1Rs) located on SPNs, in turn preventing the expression of corticostriatal long-term depression (LTD) and synaptic depotentiation (SD), whereas long-term potentiation (LTP) is enhanced [26, 27]. Notably, the alteration of corticostriatal synaptic plasticity occurs in an early time-window of brain development in mice, and is associated with changes in synaptic morphology [91]. Pharmacological manipulation of eukaryotic translation initiation factor subunit 2 alpha (eIF2α) signaling has been shown to restore corticostriatal LTD (Fig. **1**) [92]. A possible interaction between TA and eIF2a may derive from their crucial role in the cellular response to endoplasmic reticulum stress, supported by evidence in human DYT-*TOR1A* brains [93]. Accordingly, experimental evidence suggests that environmental stress, forcing striatal dopaminergic dysregulation in DYT-*TOR1A* rodent models, induces the manifestation of dystonic symptoms [94, 95]. To investigate the origin of dopaminergic dysfunction, a conditional model expressing *Tor1a(ΔE)* selectively in dopaminergic neurons has been generated. This approach demonstrated a cell-autonomous effect of the DYT1 mutation on dopamine release [96].

TA is also highly expressed in cerebellar PC and at different glutamatergic and GABAergic cerebellar synaptic inputs [97]. In fact, the loss of TA during cerebellar synaptogenesis induces developmental synaptic alterations in DYT-*TOR1A* mouse models [98].

In summary, several pieces of evidence point to a key role of the *Tor1a(ΔE)* mutation in generating synaptic dysfunction in DYT-*TOR1A* dystonia.

### 4.2 DYT-THAP1 Dystonia

DYT-*THAP1* is an early-onset generalized dystonia with predominant craniocervical involvement caused by loss-of-function mutations in the zinc finger transcription factor THAP-1 (Thanatos-associated [THAP] domain-containing apoptosis-associated protein 1) [99, 100]. Despite the ubiquitous expression of the mutated protein, DYT-*THAP1* pathogenesis is due to its role in the central nervous system, where the THAP-1 mutation induces dysregulation of gene expression mainly in the striatum and cerebellum [101]. Different rodent models of DYT6 have been generated, including KI, C54Y and null mutations of the *THAP1* gene. In the brain of a mouse model and in cell models, DYT-*THAP1* mutations cause changes in the expression profile of genes related to neurodevelopment, dopaminergic and cholinergic networks, and synaptic function, including the eIF2α signaling and its key effector ATF4 [14, 62, 101-103] (Fig. **1**). Indeed, it has been recently shown that THAP1 is involved in the control of myelination during neuronal maturation, supporting its role in the neurodevelopment [104]. Interestingly, similarly to DYT-*TOR1A* models, an increased striatal level of acetylcholine has been described in THAP1^+/-^ mice, and THAP1^+/-^ rats present behavior abnormalities and dopaminergic dysfunction [62, 105]. Furthermore, similar to DYT-*TOR1A* dystonia models, corticostriatal synaptic plasticity is disrupted in THAP1^+/-^ mice [101]. This alteration may be due to the impairment of the eIF2α pathway, representing a further pathophysiological feature shared by DYT6 and DYT1 dystonia models [92, 101]. The complete removal of the *Thap1* gene in glial and neuronal precursors causes locomotory deficit and abnormalities in the basal ganglia and in the cerebellar circuitry [103]. Indeed, THAP1 levels are important for cerebellar function, and cerebellar abnormalities may contribute to motor symptoms of DYT-*THAP1* dystonia [48, 106].

### 4.3 DYT-GNAL Dystonia

Loss-of-function mutations within the *GNAL* gene cause DYT25 dystonia [107-109]. This form of dystonia manifests mainly in adulthood as focal craniocervical or segmental dystonia, although also a childhood-onset form has been recently described [110]. The *GNAL* gene encodes the stimulatory G-protein G_αolf_, which is highly enriched in the striatum, where it couples the D1R, expressed by direct pathway SPNs, and adenosine A2A receptor (A2AR), expressed by indirect pathway SPNs, to adenylyl cyclase 5 (AC5) signaling [111]. Hence, *GNAL* loss-of-function mutations can affect both basal ganglia pathways, reinforcing the concept that a coordinated activation of both is necessary during the movement [112]. Homozygous and heterozygous genetic models of DYT25 have been generated. Since the complete removal of G_αolf_ in homozygous *Gnal*^-/-^ rodents determines anosmia and death for inability to feed and only a minor percentage of surviving mice are hyperactive, the heterozygous models are the most studied Heterozygous *Gnal*^+/-^ mice show a mild behavioral phenotype, which is worsened by systemic administration of oxotremorine, a muscarinic receptor agonist [113, 114], as observed in the DYT1 KI mice [115]. In accordance with the pattern of G_αolf_ expression, dysfunction observed in *Gnal*^+/-^ rodent models was reported in the striatum, at the postsynaptic level [113, 116]. Recent work on the *Gnal*^+/-^ mouse model reported a significant reduction of basal and stimulated cAMP levels, and a decreased ERK kinase phosphorylation activity in SPNs of the direct pathway [115] (Fig. **1**). It is well documented that the expression of physiological bidirectional plasticity at corticostriatal synapses depends on dopamine receptor-mediated transmission [23]. Recent work on a *Gnal*^+/-^ rat model reported an impairment of corticostriatal LTD, which was partially rescued by a combination of both dopamine D1R and D2R agonists, but fully rescued by antagonism of either adenosine A2A or metabotropic glutamate type 5 receptor (mGlu5R) [116-118]. Since both A2AR and mGlu5R exert antagonistic actions on striatal D2R, these observations suggest that the rescue of dopamine-dependent LTD is in fact, mediated by the disinhibition of D2R. In addition, G_αolf_ deficiency increases the sensitivity to D2R antagonism, which leads to an increased catalepsy response in the *Gnal*^+/-^ mice [119]. One of the downstream proteins in the G_αolf_ signaling cascade is Arc, a regulator of AMPA receptors (AMPAR). Biochemical studies in DYT-*GNAL* rodent models have revealed downregulation of Arc protein in the striatum, leading to an increased expression of AMPARs, a reduction of phospho-CaMKII, and an altered morphology of SPNs dendritic spines [113, 116]. In ChIs, where both G_olf_ and G_s_ are expressed [120], a D2R-induced paradoxical activity has been described, which was prevented by an A2A antagonist [62].

### 4.4 DYT-SGCE Dystonia

Mutations in epsilon(ε)-sarcoglycan (*SGCE*) gene cause DYT11 myoclonus-dystonia, the most common ‘dystonia-plus’ syndrome, also known as alcohol-responsive dystonia since the motor symptoms may improve after consuming alcohol [121, 122]. The SGCE protein is mainly expressed in the molecular layer of the cerebellum, and in the CP and GP of the basal ganglia [123]. Changes in neuronal firing patterns and gray matter volume in the GPi correlate with motor signs, explaining the effectiveness of GPi-DBS in patients [49, 67, 124, 125]. However, once again, clinical and experimental evidence support an involvement of both the basal ganglia and the cerebellum in the pathophysiology of DYT-*SGCE* dystonia [49, 126-128]. Transgenic models of DYT11, including *Sgce* conditional and KO mice, have been generated. Furthermore, experimental evidence showed that either SGCE knockdown (KD) in the cerebellum (*Sgce* KD CB), conditional KO in PC cells (*Sgce* pKO), or conditional KO in the striatum (*Sgce* sKO) causes motor deficits in mice [49, 129, 130]. Heterozygous *Sgce* KO mice show loss of corticostriatal LTD, which is restored by inhibiting adenosine A2AR [131]. The A2AR-mediated effect is probably due to an indirect action on the striatal D2R. Indeed, loss of SGCE results in decreased striatal D2R protein and enhanced dopamine metabolites levels in heterozygous *Sgce* KO mice [127, 132], in accordance with the reduction of both D2R binding and level observed in DYT-*SGCE* dystonia patients [133, 134]. The *SGCE* gene encodes a single pass transmembrane protein belonging to the dystrophin-glycoprotein complex, implicated in brain synaptic function [135]. Indeed, compelling evidence has shown that the dystrophin complex may regulate the post-synaptic machinery at inhibitory synapses [136, 137] (Fig. **1**). In fact, it localizes at GABAergic synapses, influencing the postsynaptic clustering of GABAA receptors [138]. In addition, SGCE, by mediating the link between the cytoskeleton and the extracellular matrix, influences the morphology of dopaminergic neurons [135]. As discussed in a recent review, alcohol could exert its beneficial effects in DYT11 by modulating the output from the dorsal striatum and, in turn, motor control [139]. In more detail, alcohol may act on the dopaminergic system and striatal LTD induction either directly or indirectly, through the endocannabinoid system [140]. Alternatively, alcohol effects could be due to its ability to down-regulate the tonic firing frequency of striatal cholinergic interneurons, thus lowering the levels of striatal acetylcholine [141]. However, alcohol may also act at the cerebellar level. Knockdown of *Sgce* in the cerebellum of adult mice induced the development of motor symptoms, including dystonia, which could be relieved by alcohol administration [49]. It was recently proposed that low doses of ethanol might be effective in DYT11 by normalizing an abnormal activation of Purkinje cells and dentate nucleus [142].

### 4.5 DYT/PARK-GCH1 and DYT/PARK-TH

Previous studies have described early-onset dystonia in combination with parkinsonism as one of the phenotypic hallmarks of DOPA-responsive dystonia (DRD or DYT5) [143, 144]. Thus, the use of the prefix “DYT/PARK” preceding the specific gene name to classify the different DRD syndromes was recommended [145]. Pathogenic variants in five genes, namely GTP cyclohydrolase 1 (*GCH1*), tyrosine hydroxylase (*TH*), 6-pyruvoyl tetrahydrobiopterin synthase (*PTS*), sepiapterin reductase (*SPR*), and quinoid dihydropteridine reductase (*QDPR*), involved in dopamine/tetrahydro-biopterin (BH4) biosynthesis or recycling, have been linked to DRD [145]. Mutations at the DYT5 locus in the gene coding for GCH1, the rate-limiting enzyme in the synthesis of the TH cofactor BH4, are responsible for the most frequent DYT/PARK-*GCH1* autosomal dominant forms of DRD [146-148]. The GCH1 deficiency determines a reduction of BH4, and consequently of dopamine levels, and is associated with an increase of D2R binding in both manifesting and non-manifesting carriers of the DYT/PARK-*GCH1* mutation [149, 150]. Furthermore, also the mutations in *TH itself* lead to the striatal deficit of DA and DRD. Indeed, transgenic KI mice carrying the mutation in the *GCH1* or *TH* genes have been created. The KI mouse model of DYT/PARK-*GCH1*, carrying a missense mutation in the *GCH1* gene, is characterized by a significant reduction in BH4 level and undetectable TH (Fig. **1**). This mouse model recapitulates the main clinical features of the human disorder, since striatal dopamine concentration is reduced and the local administration of L-DOPA ameliorates the motor and vocalization deficits [57, 151-153]. Despite anticholinergic therapy being supportive in DYT/PARK-*GCH1* dystonia [154], recent evidence reported only subtle changes in the morphology of striatal cholinergic interneurons in DYT/PARK-*GCH1* mice [155].

A KI mouse model of autosomal recessive DYT/PARK-*TH* forms of DRD has also been generated, by inserting a point mutation in the murine *TH* gene. This model has been well characterized and shows construct, face and predictive validity [151]. An interesting aspect emerging from this mouse model is an aberrant response to the activation of both D1R and D2R [152]. Homozygous mice show reduced enzymatic activity and immunostaining of TH, mainly in the striosomes, in addition to dystonic movements and a hypokinetic behavior consistent with a dopaminergic deficit, although no nigral degeneration was observed [156]. The loss of striatal TH manifests in the early postnatal period, at the onset of the motor symptoms [157].

## CONCLUSION

Experimental models with construct, face and predictive validity are fundamental tools to identify novel therapeutic targets [158]. To this aim, a vast effort has been made in modeling dystonia, [7, 9, 10, 60, 86]. Research on genetic animal models of different forms of dystonia is expected to provide a better understanding of common pathogenic mechanisms, and to help identify more effective treatments [60]. To date, growing evidence suggests that an aberrant neural network, involving the sensorimotor cortex, basal ganglia and cerebellum, underlies dystonia pathophysiology [3]. A great deal of evidence of synaptic dysfunction in the striatum of dystonia models has been provided, and alterations have also been reported in the cerebellum and GP, meaning that gene mutations linked to different forms of dystonia may converge on synaptic dysfunction [62, 12, 14, 15]. Indeed, animal models of monogenic dystonia represent an important tool to investigate the pathogenesis of dystonia to pinpoint common molecular mechanisms and to identify novel therapeutic targets in order to facilitate the development of disease-modifying therapies.

Despite synaptic dysfunction represents an endophenotype, since rodent models of dystonia often do not manifest an overt motor phenotype, the majority of dystonia rodent models show some level of face validity, in that they share some common alterations with patients. For example, reductions in striatal D2R and binding activity were observed in mouse models and patients of DYT-*TOR1A* and DYT-*SGCE* dystonia [86, 159, 160]. Additionally, anticholinergics are effective in the clinical treatment of DYT-*TOR1A* patients, as well as in rescuing synaptic alterations in rodent models. The validity of preclinical models offers the opportunity to identify the molecular alterations observed in rodents as potential biomarkers or therapeutic targets for clinical practice. Unfortunately, dystonia rodent models often do not show a motor phenotype, but it has been suggested that they might model a non-phenotypic carrier condition. Indeed, in many inherited disorders, the same mutation does not cause the expression of a disease phenotype in all the carriers [161]. Animal models of dystonia forms with reduced or incomplete penetrance, such as DYT-*TOR1A*, DYT-*THAP1*, DYT-*GNAL*, DYT/PARK-*GCH1,* DYT/PARK-*TH* and DYT-*SGCE*, offer the opportunity to study the pathophysiological mechanisms leading to the expression of a dystonic phenotype. Recently, the expression of a dystonic phenotype has been obtained in two models of DYT-*TOR1A* by means of an environmental trigger [94, 95]. The possibility to induce an overt motor phenotype in animal models will allow to better model the interaction between a genetic insult, causing the synaptic dysfunction, and an external event, triggering the expression of the dystonic symptoms.

## Figures and Tables

**Table 1:** Basal ganglia synaptic dysfunction in dystonia.

	**Experimental Models**	**Basal Ganglia Synaptic Dysfunction**	
		**Rodent**	**Human**
**DYT-*TOR1A***	Tor1a^+/-^ null mice [84] Tor1a ^Δgag/+^ mice [84] Conditional ΔE mice [96] Symptomatic model Tor1a^+/-^ mice and rat [94, 95]	• Reduction of striatal D2 receptor and impairment of dopaminergic system [85, 94, 95, 96]. • Impairment of the cholinergic system [63, 80, 87, 89]. • Impairment of synaptic proteins and glutamatergic release [15]. • Imbalance of corticostriatal plasticity [26, 27]. • Dysregulation of opioidergic and purinergic system [81, 83]. • Abnormal eIF2 signaling [92]. • Changes in PDE10 expression and activity in the striatum and GP [82].	• Reduction of striatal D2 receptor and binding [159, 160]. • Reduction of the connection GPe, increased inhibition of the STN and the GPi [64, 65]. • GPi-DBS ameliorate the symptoms [66, 69]. • eIF2α signaling is impaired in patients [92, 93]. • Abnormalities in the cortico-striato-pallido-thalamo cortical pathway [34].
**DYT-*THAP1***	Thap1^+/-^ rat [105] Thap1^C54Y/+^ mice [48] Thap1 null mice [103] Thap1^+/-^ mice [48]	• Cholinergic dysfunction [62, 105]. • Behavior abnormalities and dopaminergic dysfunction [105]. • Corticostriatal plasticity imbalance [101]. • Abnormal eIF2α signaling [101]. • Locomotory deficit [101]. • Decrease of dendritic arborization and spine density in SPNs [101].	• Abnormalities in the cortico-striato-pallido-thalamo cortical pathways [34, 36]. • Dopaminergic dysfunction [57]. • Reduction of striatal D2 receptor and binding [159]. • GPi-DBS ameliorate the symptoms [70]. • Dysregulation of gene expression related to synaptic function [14].
**DYT-*GNAL***	GNAL^+/-^ rats [116] GNAL^+/-^ mice [113]	• Cholinergic dysfunction [61, 62]. • Increase sensitivity of D2 receptor antagonism [119]. • Behavioral impairment [113, 119]. • Altered morphology of SPNs [113, 119]. • Impaired LTD [116, 117]. • Reduction of basal and stimulated levels of cAMP [115]. • Decreased ERK kinase phosphorylation activity [115].	• GPi-DBS ameliorate the symptoms [66, 68]. • Response to anticholinergic drugs [39].
**DYT-*SGCE***	Sgce-KO mice [130, 132] Conditional Sgce-sKO mice [129] Conditional Sgce-KD CB mice [49] Sgce^+/-^ mice [131]	• Impaired LTD [131]. • Decreased striatal D2R levels [127]. • Enhance of dopaminergic neurotransmission [132, 135]. • Behavioral impairment [130, 132]. • Abnormal nuclear envelope in the SPNs [129]. • Change of firing of the GPi neurons [49].	• Reduction of striatal D2 receptor and binding [133]. • Basal ganglia metabolic abnormalities [134]. • GPi-DBS ameliorate the symptoms [124]. • GP activity correlates with motor signs [67]. • Putaminal gray matter volume [125]. • Amelioration of symptoms after ethanol ingestion [139].
**DYT/** **PARK-*GCH1*** **DYT/** **PARK-*TH***	GCH1 knockin mice [153] DRD knockin mice [151]	• Reduction of BH4 and undetectable levels of TH [153]. • Subtle alteration of the cholinergic system [155]. • Reduced activity and immunostaining of TH, no nigral degeneration [151, 156]. • Dystonic movements, hypokinetic behavior that ameliorates with L-DOPA [156]. • Aberrant response to activation of both D1R and D2R [151].	• Reduction of BH4 and dopamine levels [149, 150]. • Abnormal dopaminergic system and parkinsonism [148]. • Response to L-DOPA and anticholinergic therapy [154]. • Increase of D2R binding [150]. • No nigral cell loss [148].

**Table 2:** Cerebellar synaptic dysfunction in dystonia.

	**Experimental Models**	**Cerebellar Synaptic Dysfunction**	
		**Rodent**	**Human**
**DYT-*TOR1A***	Tor1a^+/-^ null mice [84] Tor1a^Δgag/+^ mice [84]	• Alteration of dendritic spines of PC [44, 45]. • Changes in the activity of PC and DN neurons [46, 47]. • Cerebellar synaptogenesis altered [98]. • Cerebellar knockdown of Tor1a gene results in dystonic movements [45].	• Increased metabolic cerebellar activity [34]. • Structural lesions in cerebellar outflow pathways [36]. • Abnormality cerebello-thalamo-cortical circuitry [34]. • DBS ameliorates the symptoms [36, 38].
**DYT-*THAP1***	Thap1^+/-^ mice [48] Thap1 null mice [103]	• Structural abnormalities of the DN cells [48]. • PC and DN neurons have abnormal firing patterns [106]. • Locomotory deficit [103].	• Abnormalities in the cerebello-thalamo-cortical and pathway [34, 36]. • Structural lesions in cerebellar outflow pathways [36]. • DBS ameliorates the symptoms [36].
**DYT-*GNAL***	GNAL^+/-^ mice [113]	• Abnormal cerebello-thalamic pathway [50].	• Cerebellar rTMS under investigation [39]
**DYT-*SGCE***	Sgce-KO mice [130] Sgce pKO mice [130] Conditional Sgce-KD CB mice [49]	• Firing rate reduction of PC and DN cells [49]. • Dysregulated cerebellar gene expression interferes with motor learning function [123, 130]. • Knockdown of cerebellar SGCE produces motor symptoms [49]. • Abnormal nuclear envelopes of PC cells [130].	• Metabolic abnormalities [134, 135]. • Abnormalities in cerebellar structure and function [128]. • Dysfunction of the cerebello-thalamic pathway [126].
**DYT/** **PARK-*GCH1*** **DYT/** **PARK-TH**	nd	nd	nd

## LIST OF ABBREVIATIONS

D2R: Dopamine 2 Receptor
DBS: Deep Brain Stimulation
DN: Deep Nuclei
GP: Globus Pallidus
HD: Huntington's Disease
LTP: Long-term Potentiation
PC: Purkinje Cells
PD: Parkinson’s Disease
PET: Positron Emission Topography
rTMS: Repetitive Transcranial Magnetic Stimulation
SD: Synaptic Depotentiation
SN: Substantia Nigra
STN: Subthalamic Nucleus
